# Activation of PPARγ in Myeloid Cells Promotes Lung Cancer Progression and Metastasis

**DOI:** 10.1371/journal.pone.0028133

**Published:** 2011-12-01

**Authors:** Howard Li, Amber L. Sorenson, Joanna Poczobutt, Jay Amin, Teresa Joyal, Timothy Sullivan, Joseph T. Crossno, Mary C. M. Weiser-Evans, Raphael A. Nemenoff

**Affiliations:** 1 Division of Pulmonary Sciences and Critical Care Medicine, Department of Medicine, University of Colorado Anschutz Medical Campus, Aurora, Colorado, United States of America; 2 Division of Renal Diseases and Hypertension, Department of Medicine, University of Colorado Anschutz Medical Campus, Aurora, Colorado, United States of America; 3 Cardiovascular Pulmonary Research Laboratory, Department of Medicine, University of Colorado Anschutz Medical Campus, Aurora, Colorado, United States of America; 4 Veterans Affairs Medical Center, Denver, Colorado, United States of America; Technische Universität München, Germany

## Abstract

Activation of peroxisome proliferator-activated receptor-γ (PPARγ) inhibits growth of cancer cells including non-small cell lung cancer (NSCLC). Clinically, use of thiazolidinediones, which are pharmacological activators of PPARγ is associated with a lower risk of developing lung cancer. However, the role of this pathway in lung cancer metastasis has not been examined well. The systemic effect of pioglitazone was examined in two models of lung cancer metastasis in immune-competent mice. In an orthotopic model, murine lung cancer cells implanted into the lungs of syngeneic mice metastasized to the liver and brain. As a second model, cancer cells injected subcutaneously metastasized to the lung. In both models systemic administration of pioglitazone increased the rate of metastasis. Examination of tissues from the orthotopic model demonstrated increased numbers of arginase I-positive macrophages in tumors from pioglitazone-treated animals. In co-culture experiments of cancer cells with bone marrow-derived macrophages, pioglitazone promoted arginase I expression in macrophages and this was dependent on the expression of PPARγ in the macrophages. To assess the contribution of PPARγ in macrophages to cancer progression, experiments were performed in bone marrow-transplanted animals receiving bone marrow from Lys-M-Cre+/PPARγ^flox/flox^ mice, in which PPARγ is deleted specifically in myeloid cells (PPARγ-Mac^neg^), or control PPARγ^flox/flox^ mice. In both models, mice receiving PPARγ-Mac^neg^ bone marrow had a marked decrease in secondary tumors which was not significantly altered by treatment with pioglitazone. This was associated with decreased numbers of arginase I-positive cells in the lung. These data support a model in which activation of PPARγ may have opposing effects on tumor progression, with anti-tumorigenic effects on cancer cells, but pro-tumorigenic effects on cells of the microenvironment, specifically myeloid cells.

## Introduction

Lung cancer is the leading cause of cancer deaths in both men and women worldwide, and survival rates remain low [1]. A principal reason is that many patients present with advanced disease and metastases at the time of diagnosis. Therefore, translational studies designed to identify pharmaceuticals that inhibit metastasis are essential to improving clinical outcomes. Although genetic changes in cancer cells drive tumor initiation, the tumor microenvironment plays a critical role in tumor progression and metastasis [2]. Interactions between tumor cells and cells in the tumor microenvironment (e.g. vascular cells, immune cells, fibroblasts) control tumor angiogenesis and can promote a more aggressive phenotype. These cell-cell interactions are mediated through cytokines and growth factors initially produced by the tumor cells, which result in immune and vascular cell recruitment. The role of the tumor microenvironment in lung cancer has not been as extensively studied as in other types of cancer, such as breast and prostate, at least in part because of the lack of good animal models. Chemical carcinogenesis models have been important in studying tumor initiation, but the resulting tumors are usually adenomas which do not metastasize. Genetic mouse models have also been employed, but although these form adenocarcinomas, they are often weakly metatastic [3]. Studies with human lung cancer cell lines have used xenograft models in which tumor cells are inoculated subcutaneously into immunocompromised rodents. Thus the environment in which the primary tumor develops is not the lung, and the full role of immune cells on tumor progression cannot be assessed. We have therefore developed an orthotopic model in which mouse tumor cells derived from lung tumors in C57BL/6 mice [4] are directly injected into lungs of syngeneic mice [5], allowing an assessment of tumor progression and metastasis in immunocompetent animals. This provides a clinically relevant system in which to test the efficacy of new strategies/pharmaceuticals designed to target lung cancer progression and metastasis.

Peroxisome proliferator-activated receptor-γ (PPARγ) is a member of the nuclear hormone receptor superfamily of ligand-activated transcription factors [6]. The classic pathway of PPARγactivation involves binding as a heterodimer with the retinoic acid X receptor to specific DNA sequences in the promoters of target genes. Ligand binding causes a conformational change, resulting in the release of co-repressors and the binding of co-activators. PPARγ has also been shown to bind to other transcription factors resulting in transrepression [6]. Endogenous PPARγ activators include polyunsaturated fatty acids and eicosanoids, while synthetic activators of PPARγ include the thiazolidinediones (TZDs), such as rosiglitazone and pioglitazone [7]. It has been well documented that PPARγ activation plays a critical role in adipocyte activation and differentiation. Recently, however, PPARγ has also been implicated in regulating multiple types of cancer, including lung cancer. Analysis of human lung tumors has reported that decreased expression of PPARγ is correlated with a poor prognosis [8]. Importantly, a retrospective study examining cancer incidence in diabetic patients using TZDs demonstrated a 33% reduction in lung cancer risk [9], with an even more dramatic reduction in African-American diabetic patients (75%). This decreased risk was specific for lung cancer, with no protective effect observed for prostate or colon cancer. Preclinical studies from our laboratory demonstrated that activation of PPARγ in human non-small cell lung cancer cell (NSCLC) lines inhibited transformed growth and invasiveness, and promoted a more differentiated phenotype [10], [11]. Furthermore targeted overexpression of PPARγ in mice to distal type II alveolar epithelial cells had chemopreventive effects by inhibiting the initiation of lung tumors [12]. Collectively, these studies suggest that targeting PPARγ may have important chemopreventive applications for lung cancer. However, the role of PPARγ activation by systemic administration of TZDs in regulating tumor progression and metastasis of lung cancer has not been well studied. In fact, the retrospective clinical study which showed decreased incidence of lung cancer in diabetics treated with TZDs excluded individuals who had a pre-existing diagnosis of cancer [9]. The goal of our study was to determine the effect of systemic PPARγ activation on lung cancer progression and metastasis using the TZD pioglitazone in our orthotopic immunocompetent model. In contrast to our original hypothesis that pioglitazone would exert protective effects on lung cancer metastasis, here we report the unexpected findings that systemic administration of pioglitazone accelerates the rate of lung tumor progression and metastasis, and this is mediated through activation of PPARγin myeloid cells.

## Results

### Pioglitazone-fed mice exhibit increased lung cancer progression and metastasis

**Figure 1:**
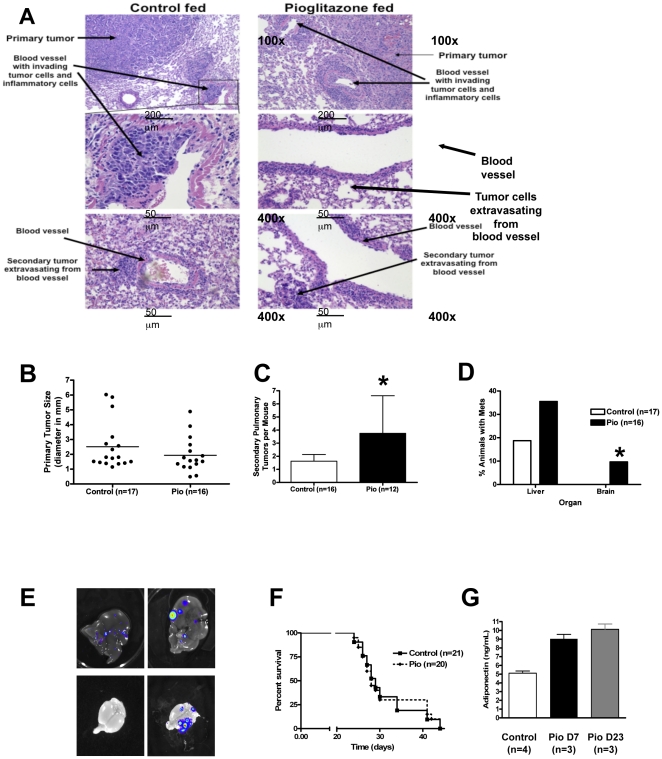
Pioglitazone accelerates CMT/167 tumor progression and metastasis in an orthotopic mouse model of NSCLC. Wild-type C57BL/6 mice were fed either normal chow or chow impregnated with pioglitazone (0.05%) for 1 week prior to tumor cell implantation and throughout the course of the experiment. CMT/167-luc cells (10^5^) were orthotopically injected into the left lung as described in the Methods section. Lungs, livers, and brains were harvested 25–30 days post-injection. ***A*** *.* Hematoxylin-eosin stained sections of tumor-bearing lungs. Arrows indicate primary or secondary lung tumors, as well as tumors intravasating into or extravasating out of a blood vessel. ***B*** *.* Quantitation of primary tumor diameter in control (n = 17) and pioglitazone-treated (n = 16) animals measured using digital calipers. ***C.*** Number of secondary pulmonary tumors in C57BL/6 mice fed either normal chow or pioglitazone chow were determined by quantifying tumors in hematoxylin-eosin stained sections through the middle of the lungs. Data are means and s.d. of counts from 12-16 animals in each group. ***D.*** Percentage of C57BL/6 mice fed either normal chow (n = 17) or pioglitazone-impregnated chow (n = 16) with liver and brain metastases. Liver and brain metastases were identified by *ex vivo* bioluminescent imaging of organs at the time of sacrifice and were confirmed by histology. ***E.*** Representative bioluminescent images of liver metastases in mice fed either normal chow (upper left panel) or pioglitazone-impregnated chow (upper right panel). Representative bioluminescent images of brains from mice fed either normal chow (lower left panel) or pioglitazone-impregnated chow (lower right panel). ***F*** *.* Kaplan-Meier survival curve of C57BL/6 mice injected with CMT/167-luc cells fed either control or pioglitazone-impregnated chow. ***G.*** Mean serum concentrations of adiponectin in C57BL/6 mice fed either normal chow or pioglitazone-impregnated chow. For all graphs *P<0.05 vs Control.

To assess the effect of systemically administered pioglitazone on lung cancer progression and metastasis in immune-competent mice we used CMT/167 cells, a lung adenocarcinoma cell line derived from C57BL/6 mice [13]. We have previously shown that injection of these cells into the lungs of syngeneic C57BL/6 mice results in a primary tumor, which subsequently progresses to form secondary pulmonary tumors, and metastasizes to the lymph nodes and distant organs [5]. In vitro experiments demonstrated that pioglitazone inhibits invasiveness of these cells (Figure S1), and promotes a more differentiated phenotype in 3-dimensional Matrigel cultures (Figure S1), consistent with what we have observed with PPARγ activation in human NSCLC [10]. To examine the systemic role of PPARγ in vivo wild-type C57BL/6 mice were placed on control or pioglitazone-impregnated chow 7 days prior to cancer cell injections and throughout the course of the experiment. After 7 days, cancer cells stably expressing luciferase (CMT/167-luc) suspended in Matrigel (BD Biosciences) were injected through the rib cage into the left lobe of mice as previously described [5]. Primary tumor formation was analyzed 3 days after injections by in vivo bioluminescent imaging; mice that did not show the development of a primary tumor were removed from the study. At various times after injections, mice were sacrificed and lungs, heart, liver, and brains removed for ex vivo bioluminescence imaging and histological analysis. Examination of H&E stained lung sections revealed large primary tumors and the presence of cancer cell intravasation into blood vessels adjacent to primary tumors as well as cancer cell extravasation from blood vessels in other lung lobes (Figure 1A). Growth of the primary tumor was not significantly different between the two groups of mice (Figure 1B). In contrast, pioglitazone-fed mice exhibited markedly increased numbers of secondary pulmonary metastases, which were determined by counting number of tumors in H&E lung sections (Figure 1C). Incidence of liver and brain metastases was quantified at 25–30 days post-injection by ex vivo bioluminescent imaging. The incidence of liver metastases in pioglitazone-treated mice was twice that of the control group (Figure 1D,E). In the pioglitazone-treated group approximately 10% of the animals developed brain metastases, while no brain metastases were detected in the control group (Figure 1D,F). Overall survival was not different in the two groups of mice (Figure 1G). To validate that mice fed pioglitazone-impregnated chow received the drug and that PPARγ was being activated, serum was collected and adiponectin levels were measured by ELISA. As has been previously published [14], serum adiponectin levels were increased within 7 days of pioglitazone administration, and remained elevated after 23 days (Figure 1H).

### Systemically administered pioglitazone increases the numbers of arginase I-positive macrophages within tumors

**Figure 2:**
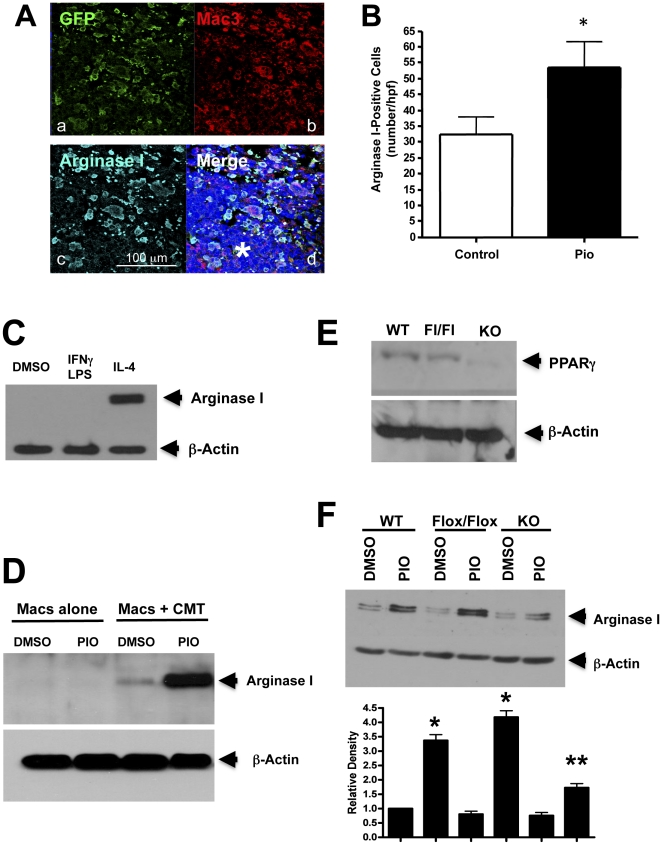
Effect of pioglitazone on cells in the tumor microenvironment. ***A.*** Representative immunofluorescent staining for GFP (a), Mac3 (b), arginase I (c), and the overlay of all three with DAPI (d) in a primary tumor and surrounding tissue 16 days after cancer cell injection from a pioglitazone-treated recipient mouse that received a bone marrow transplant from a UBI-EGFP transgenic donor. The asterisk indicates the tumor. ***B.*** Quantitation of arginase I-positive cells within the tumors 16 days after injection. Data are means and s.e.m. of counts from 11–13 animals in each group, using 1–2 slides per animal and counting 4 random fields per slide. *P<0.05 vs Control. ***C.*** Bone marrow-derived macrophages isolated as described in the Methods section were stimulated for 18 hours with either IFNγ/LPS or IL-4 and analyzed for expression of arginase I. ***D.*** Identical cells from (C) were grown alone or in co-culture with CMT/167 cells for 3 days in the absence or presence pioglitazone (10 µM). Cell lysates were immunoblotted for arginase I. ***E.*** Whole cell lysates from WT, PPARγ^flox/flox^ (Fl/Fl), or PPARγ-Mac^neg^ (KO) macrophages were analyzed for PPARγexpression. ***F.*** WT, PPARγ^flox/flox^, or PPARγ-Mac^neg^ macrophages were co-cultured with CMT/167 cells in the presence or absence of pioglitazone (10 µM). Cell lysates were analyzed for arginase I expression. Densitometry measurements showing normalized levels relative to control are displayed below the western blot (average of three independent experiments). The densitometric analysis was performed using ImageJ software (NIH, Bethesda, MD) as described in the Methods section. *P<0.05 vs WT control, **P<0.05 vs WT PIO. For all Westerns, b-actin was used as a loading control.

Recruitment of bone marrow–derived cells, and in particular monocytes/macrophages, contributes to the tumor microenvironment and is associated with more aggressive, malignant tumors [15]. To characterize the effects of systemic PPARγ activation induced by pioglitazone on bone marrow cell recruitment and tumor progression, WT mice received bone marrow transplants from UBI-EGFP/B6 transgenic donors, which express EGFP in all cells. After allowing 6 weeks for recovery, animals were placed on appropriate chow for 7 days, and then injected with CMT/167-luc cells. Animals were maintained on either pioglitazone-impregnated or control chow throughout the course of the experiment. Lung sections were examined for GFP, Mac3 and arginase I expression by triple immunofluorescence to verify recruitment of bone marrow-derived macrophages and to determine if recruited macrophages are polarized to express an alternative M2, anti-inflammatory phenotype. Both groups of mice exhibited notable numbers of GFP(+) Mac3(+) cells surrounding the tumor (Figure 2A; representative image from pioglitazone-fed lung section), indicating that the majority of the recruited bone marrow cells are macrophages. In addition, we found that the vast majority of arginase I-positive cells within and surrounding tumors were Mac3(+) macrophages (Figure 2A). Tumor-associated macrophages (TAMs) were quantified by counting Mac3(+) cells in primary tumors of both groups of mice. Although no differences in the overall number of macrophages surrounding primary lung tumors were found between the two groups of mice used in our orthotopic lung experiment (data not shown), the number of arginase I-positive macrophages within primary tumors was markedly increased in mice fed pioglitazone-impregnated chow 16 days after implantation of tumor cells (Figure 2B).

### Pioglitazone promotes an “M2” pro-tumorigenic macrophage phenotype in co-cultures of cancer cells and macrophages

To determine whether PPARγ plays a role in macrophage M2 polarization, bone marrow cells isolated from wild type C57BL/6 male mice were grown in the presence of recombinant M-CSF to promote differentiation into macrophages [16]. These cells have the morphology of macrophages, are >95% F4/80 positive, and do not express notable levels of either iNOS or arginase I, markers of M1 and M2 macrophage phenotypes respectively [17]. Stimulation of these cells with IL-4 induced arginase I expression, consistent with an M2 phenotype (Figure 2C). To examine the effects of pioglitazone on interactions between cancer cells and macrophages, bone marrow-derived macrophages were co-cultured with CMT/167 cells for three days in the absence or presence of pioglitazone (10 µM) using Transwells which allow diffusible mediators to act on each cell type. CMT/167 cells selectively induced expression of arginase I in macrophages (Figure 2D), with no effect on iNOS expression (not shown). Pioglitazone as a sole agent did not affect expression of either arginase I or iNOS. However, arginase I expression was enhanced in co-cultures treated with pioglitazone (Figure 2D).

To define the contribution of PPARγ in macrophages to induction of arginase I, bone marrow-derived macrophages were isolated from Lys-M-Cre×PPARγ^flox/flox^ mice, in which PPARγ is selectively deleted in myeloid lineages (PPARγ-Mac^neg^), or control mice (PPARγ^flox/flox^) mice. Expression of PPARγ was undetectable in macrophages from PPARγ-Mac^neg^ mice, while WT and PPARγ^flox/flox^ had comparable levels of expression (Figure 2E). Importantly, co-cultures of CMT/167 cells with PPARγ-Mac^neg^ macrophages resulted in lower levels of arginase I expression in these macrophages compared to control macrophages (Figure 2F). These data indicate that activation of PPARγ in macrophages collaborates with signals from cancer cells to promote the M2 phenotype. It should be noted that pioglitazone still modestly increased arginase I expression in the PPARγ-Mac^neg^ macrophages, suggesting some contribution of PPARγ-independent “off-target” effects.

### Targeted deletion of PPARγ in macrophages inhibits metastasis

**Figure 3:**
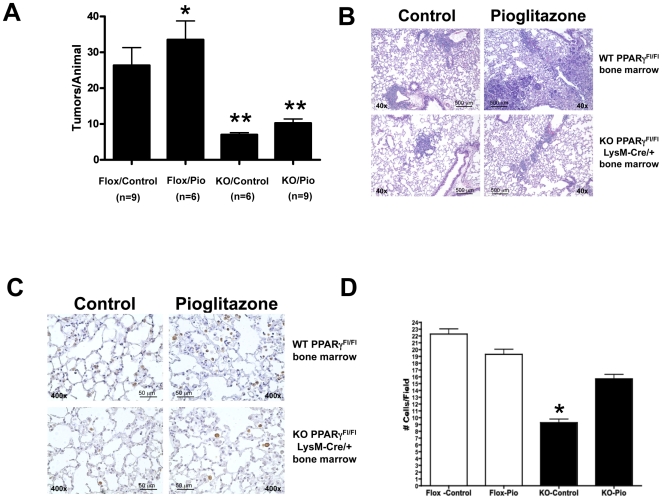
Targeted deletion of PPARγin macrophages inhibits lung cancer metastasis in an orthotopic mouse model of non-small cell lung cancer. Following lethal irradiation, WT C57BL/6 mice received bone marrow from either PPARγ-Mac^neg^ (KO) or PPARγ^flox/flox^ (Flox) donor mice as described in the “Methods” section. After 5 weeks recovery to allow engraftment, mice were placed on either pioglitazone-containing chow or control chow for 1 week prior to tumor cell implantation and throughout the course of the experiment. Animals were injected with 10^5^ CMT/167-luc cells orthotopically as in Fig. 1. Four weeks after cancer cell inoculation, animals were imaged by bioluminescence and sacrificed. ***A.*** The number of secondary lung tumors was quantitated by examination under a dissecting microscope. Tumors were counted by two independent blinded observers. Data are means and s.e.m. of counts from 6–9 animals in each group. Mice receiving PPARγ-Mac^neg^ bone marrow had significantly fewer numbers of secondary lung tumors than mice receiving PPARγ^flox/flox^. *P<0.05 vs Flox control. **P<0.05 vs Flox control. ***B.*** Representative histology (H&E) is shown for secondary lung tumors from all 4 groups of animals. ***C.*** Tumor-bearing lung sections from WT PPARγ^flox/flox^ or PPARγ-Mac^neg^ mice were immunohistochemically stained for arginase I (brown reaction color). Representative images are shown of lungs from all 4 groups of animals. ***D.*** The number of arginase I-positive cells was quantitated by two independent blinded observers. Data are means and s.e.m. of counts from 6–9 animals in each group. Lungs from PPARγ-Mac^neg^ mice on control chow showed a statistical decrease in arginase I positive cells; pioglitazone increased the number of these cells to levels seen in PPARγ^flox/flox^ mice. *P<0.05 vs Flox control.

To assess the role of PPARγ in macrophages in vivo, we performed bone marrow transplants, in which WT mice received bone marrow transplants from either PPARγ-Mac^neg^ or control PPARγ^flox/flox^ donors. Six weeks after transplantation, animals were placed on pioglitazone-impregnated or control chow for 7 days, followed by implantation of 10^5^ CMT/167-luc cells into the lung. Animals were maintained on either pioglitazone-impregnated or control chow until they were sacrificed 4 weeks after tumor implantation. Secondary pulmonary tumors were quantified by counting visible metastases under a dissecting microscope and confirmed by histology. As shown in Figure 3A, the number of secondary pulmonary tumors was greatly inhibited in all of the mice receiving bone marrow from PPARγ-Mac^neg^ mice. Pioglitazone increased secondary lung tumors in control mice, consistent with our findings in untransplanted mice. However, pioglitazone did not significantly increase the number of lung metastases in mice transplanted with bone marrow from PPARγ-Mac^neg^ mice (Figure 3A). Representative histology of the secondary lung tumors is shown in Figure 3B. Average size of the metastases was not significantly different in any of the four groups (data not shown). We examined arginase I positive macrophages in the lungs of tumor bearing animals. Pioglitazone did not alter the number of arginase I-positive cells in control PPARγ^flox/flox^ lungs. However, lungs from PPARγ-Mac^neg^ mice on control chow showed a statistical decrease in arginase I positive cells; pioglitazone increased the number of these cells to levels seen in PPARγ^flox/flox^ mice (Figure 3C,D). In all cases the great majority of arginase I positive cells stained positive for macrophage markers (data not shown).

**Figure 4:**
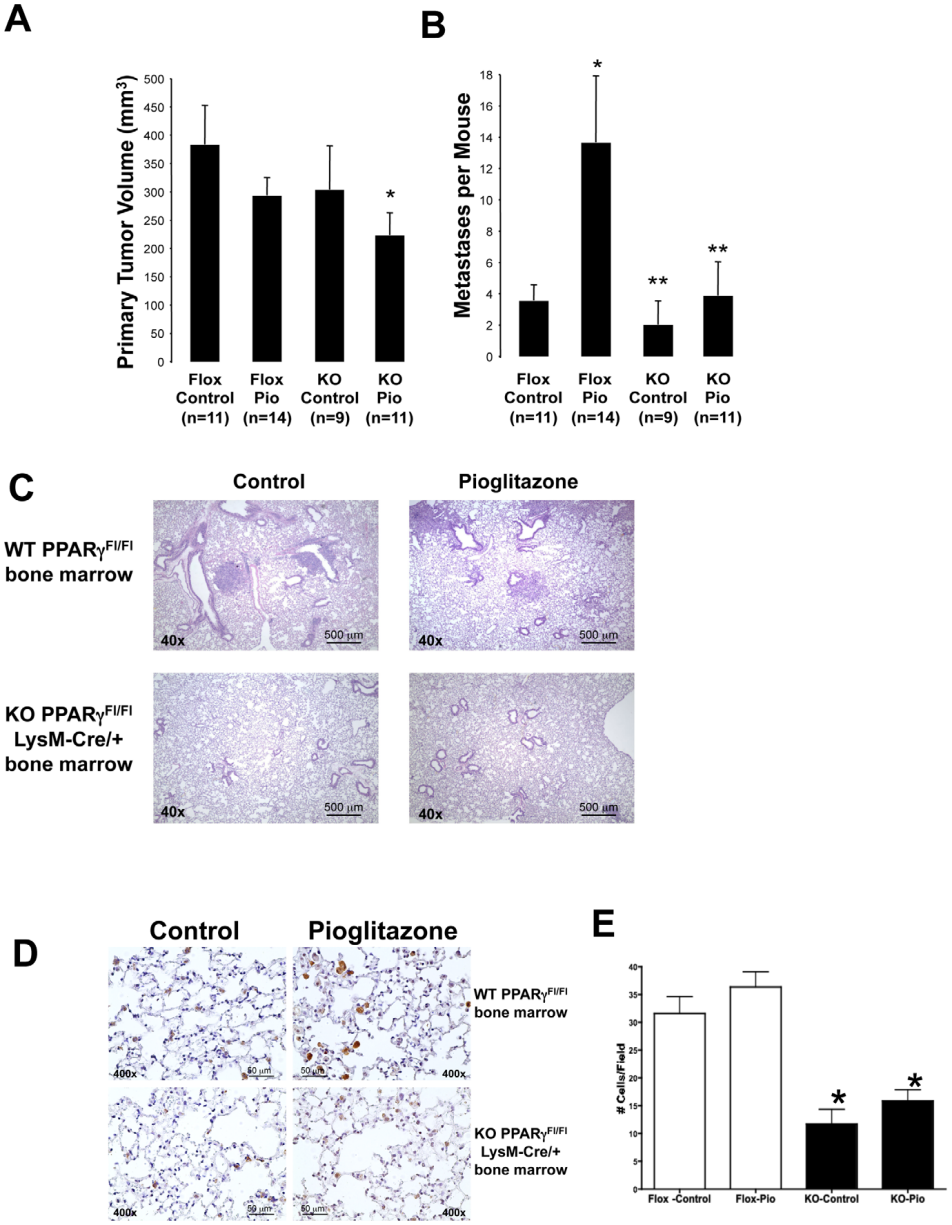
Targeted deletion of PPARγin Macrophages inhibits lung cancer metastasis in a subcutaneous flank model of murine non-small cell lung cancer. Following lethal irradiation, WT C57BL/6 mice received bone marrow from either PPARγ-Mac^neg^ (KO) or PPARγ^flox/flox^ (Flox) donor mice as described in the “Methods” section. After 5 weeks recovery to allow engraftment, mice were placed on either pioglitazone-containing chow or control chow for 1 week prior to tumor cell implantation and throughout the course of the experiment. Animals were then injected with 10^5^ CMT/167-luc cells subcutaneously. Animals were imaged by bioluminescence, and sacrificed 4 weeks after cancer cell inoculation. ***A.*** Primary tumor volumes in all the mice were measured using digital calipers. Data are means and s.e.m. of counts from 9–14 animals in each group. *P<0.05 vs Flox Control. ***B.*** Incidence of lung metastasis was quantitated by examination under a dissecting microscope. Tumors were counted by two independent blinded observers. Data are means and s.e.m. of counts from 9–14 animals in each group. Pioglitazone increased incidence of metastasis in WT mice, but not in mice receiving PPARγ-Mac^neg^ bone marrow. *P<0.05 vs Flox Control. **P<0.05 vs Flox Pio. ***C.*** Representative histology is shown for lung metastases from all 4 groups of animals. ***D.*** Tumor-bearing lung sections from WT or PPARγ-Mac^neg^ mice were immunohistochemically stained for arginase I (brown reaction color). Representative images are shown of lungs from all 4 groups of animals. ***E.*** The number of arginase I-positive cells was counted by two independent blinded observers. Data are means and s.e.m. of counts from 9–14 animals in each group with one section per animal and 4 random fields per slide. The number of arginase I-positive cells was significantly decreased in PPARγ-Mac^neg^ mice, both under control conditions and in the presence of pioglitazone. *P<0.05 vs Flox Control.

To confirm the findings in our orthotopic model, we employed a second model in which tumor cells were implanted subcutaneously in the flanks of C57BL/6 mice fed either normal or pioglitazone-impregnated chow. We used WT C57BL/6 mice transplanted with bone marrow from either PPARγ-Mac^neg^ mice or control PPARγ^flox/flox^ mice. Six weeks after transplantation, animals were placed on pioglitazone-impregnated or control chow for 7 days, followed by implantation of 10^5^ CMT/167-luc cells into the flank. Animals were maintained on either pioglitazone-impregnated or control chow until they were sacrificed 4 weeks after tumor implantation. Primary tumor size was measured with digital calipers and lung metastases were quantified by counting visible metastases under a dissecting microscope and confirmed by histology. As shown in Figure 4A, primary tumor volume was similar in PPARγ^flox/flox^ mice in the presence or absence of pioglitazone, as well as in PPARγ-Mac^neg^ mice on control chow; PPARγ-Mac^neg^ mice receiving pioglitazone exhibited a modest decrease in primary tumor size. Importantly and similar to our orthotopic model, pioglitazone markedly increased lung metastases in PPARγ^flox/flox^ mice compared to control chow-fed PPARγ^flox/flox^ mice (Figure 4B). In contrast, pioglitazone failed to significantly increase the number of lung metastases in PPARγ-Mac^neg^ mice (Figure 4B). Representative histology of the lung metastases is shown in Figure 4C. Average size of the metastases was not significantly different in any of the four groups (data not shown). Overall, we have not observed a correlation between primary tumor size and metastasis in any of the models we have studied. In addition, and similar to the orthotopic model, pioglitazone did not alter the number of arginase I-positive cells in the lungs of PPARγ^flox/flox^ mice. However, the number of arginase I-positive cells was significantly decreased in PPARγ-Mac^neg^ mice, both under control conditions and in the presence of pioglitazone (Figure 4D,E). Finally, to determine if effects on metastasis were specific to pioglitazone, experiments were repeated using chow impregnated with rosiglitazone, another TZD. Exposure to rosiglitazone showed similar increases in incidence of metastasis, but no change in primary tumor volume (Figure S2).

## Discussion

Non-small cell lung carcinoma (NSCLC) accounts for the majority of cases of lung cancer, which is the leading cause of cancer deaths worldwide. Novel therapeutic approaches to the treatment of lung cancer are urgently needed. Based on retrospective clinical studies [9], there is considerable interest in the use of TZDs as potential chemopreventive or chemotherapeutic agents in the treatment of lung cancer. This is supported by extensive studies demonstrating the effects of PPARγ activation in cancer cells on inhibition of transformed growth [18], induction of apoptosis [19], [20], and promotion of differentiation [10], [21]. In addition, PPARγ activators have been shown to inhibit tumor initiation in a chemical carcinogenesis model [22]. However, in contrast to cancer initiation, which is largely mediated through alterations in transformed epithelial cells, tumor progression and metastasis involves critical interactions between the tumor and the microenvironment. In the series of experiments we report here, systemic administration of pioglitazone to mice accelerated tumor progression and metastasis in two independent models of non-small cell lung cancer. This was reflected in increases in both the incidence and number of distant organ metastases that did not correlate to primary tumor size. Importantly and contrary to what was predicted, systemic administration of pioglitazone exerted no survival benefits compared to control mice.

There are several potential reasons for these seemingly unanticipated findings. First, as opposed to studies on human NSCLC, our studies used murine lung cancer cells, which could behave differently than human NSCLC cells. However, our findings do not support this. Activation of PPARγin CMT/167 cells inhibited invasiveness and promoted a more differentiated phenotype in 3D culture (Supporting Information S1), similar to what we have observed in human NSCLC lines [10]. Instead, we propose that the effects of pioglitazone on the acceleration of tumor progression and metastasis are mediated largely through effects on the tumor microenvironment. In fact, mice with a targeted deletion of PPARγ in myeloid lineages showed markedly fewer secondary lung tumors in our orthotopic model, and fewer lung metastases in our flank model. Furthermore pioglitazone failed to increase pulmonary tumors in both knockout models. These data to our knowledge are the first demonstration of an important role of PPARγ in the tumor microenvironment on tumor progression and metastasis. Moreover, based on our in vitro studies we propose that PPARγ in macrophages is critical for the conversion of macrophages into an alternatively activated phenotype in the presence of cancer cells which has been shown to promote metastasis [23]. Co-culture of WT macrophages with cancer cells resulted in induction of arginase I expression, a classical marker of alternatively activated macrophages, with further increases in expression observed in the presence of pioglitazone; this was markedly blunted if macrophages deficient in PPARγ were used for co-culture. Furthermore, in both metastasis models, the number of arginase I-positive macrophages in the lung was significantly decreased in mice with a targeted deletion of PPARγ in myeloid cells compared to controls indicating there is a strong correlation between macrophage-specific PPARγ activation and metastasis progression.

Evidence from clinical and experimental studies indicates macrophages promote solid tumor progression and metastasis. Macrophages are educated by the tumor microenvironment, so that they adopt a trophic role that facilitates angiogenesis, matrix breakdown and tumor cell motility, all of which are elements of the metastatic process. There is evidence that tumor-associated macrophages (TAMs) are alternatively activated, exhibiting upregulated expression of intracellular enzymes such as arginase I [17]. Previous reports have shown that arginase I production in the tumor microenvironment by mature myeloid cells inhibits T-cell receptor expression and antigen-specific T-cell responses [24]. Similarly, tumor-infiltrating and tumor-educated dendritic cells have been shown to suppress T cell responses through arginase I [25], [26]. Murine ovarian cancer vascular leukocytes also require arginase I activity for T cell suppression [27], and polymorphonuclear granulocyte arginase has been shown to impair NK cell function [28]. Collectively, these reports suggest arginase I plays a key role in the suppression of both the adaptive and innate immune system thus promoting tumor progression.

The mechanisms by which tumors educate macrophages in the tumor microenvironment and the role of PPARγ in this interaction remain poorly understood. Here we show that in vitro, CMT/167 cells induce the expression of arginase I in bone marrow-derived macrophages. Moreover, treatment with pioglitazone during the co-culture period further enhances the expression of arginase I in bone marrow-derived macrophages. Thus, cultured non-small cell lung cancer cells promote macrophage differentiation toward a phenotype that resembles the alternatively activated, pro-tumorigenic state of TAMs. This switch is not dependent on cell-cell contact, suggesting cancer cells produce diffusible factors that induce phenotypic changes in macrophages. Our in vivo findings of fewer arginase I-positive macrophages tumor-bearing lungs of mice with targeted deletion of PPARγ in myeloid cells are consistent with the in vitro findings and point to a key role for PPARγ in the innate immune system.

While many articles indicate anti-tumorigenic effects of TZDs, earlier studies have also demonstrated conflicting effects of TZDs in colon carcinogenesis. These agents inhibit colon tumor growth in a variety of immunocompromised xenograft models [29]. However, in a genetic model of colon cancer, the APC^min^ mouse, TZDs promoted tumor progression [30], [31]. This is in contrast to studies looking at tumors initiated by azoxymethane, which are inhibited by TZDs [32]. Although the basis for these disparate effects has not been established, it appears that activation of PPARγ may have opposing effects on cancer initiation vs progression in colon cancer, similar to our results in lung cancer. It should be noted that TZDs including pioglitazone have also been shown to act through “off-target” pathways [33]. While our data using genetic deletion strongly support a model in which effects in the TME are mediated through “on-target” activation of PPARγ, these studies do not exclude a role for off-target effects. In particular, pioglitazone inhibited primary tumor growth in PPARγ-Mac^neg^ mice (Figure 4). This could be a result of selective effects on tumor cells, but could also involve PPARγ-independent effects.

These findings have potential implications for patients taking PPARγ agonists, such as the TZD class of anti-diabetic agents. As mentioned above, the retrospective study showing chemopreventive effects of TZDs excluded patients who had cancer at the time that treatment was initiated [9]. However, based on this study and others, positive effects on chemoprevention of lung cancer may not ensure similar positive outcomes in the therapeutic treatment of patients with existing or prior lung cancer. Our data suggest that the net effects of an agent such as pioglitazone on tumor progression will be a balance of anti-tumorigenic effects on the cancer cells, and potentially pro-tumorigenic effects on cells of the microenvironment.

## Materials and Methods

### Animals

Wild type C57BL/6 mice were maintained in the Center for Comparative Medicine at the University of Colorado Anschutz Medical Campus. All procedures were performed under protocols approved by the Institutional Animal Care and Use Committee at the University of Colorado Denver (Protocols 06110(12)1E, 06110(06)1E and B-06110(07)1C)). For bone marrow transplant, transgenic UBI-EGFP donor mice on a C57BL/6 background were sacrificed, femurs and tibias were aseptically removed, and bone marrow obtained by aspiration. Cells were suspended in sterile HBSS+1% fetal calf serum. Recipient WT mice were irradiated (900–1,200 RAD split doses) by X-ray source at 6 wks of age. One hour following the second dose, isoflurane-anesthetized, irradiated recipients were injected with donor marrow via retro-orbital injection (2×10^6^ bone marrow-derived cells/mouse). Mice were allowed to recover and fully engraft donor bone marrow for 6 weeks prior to experimentation. UBI-EGFP transgenic mice were used as donors to track bone marrow–derived cells by GFP expression. Separate experiments were performed in which WT mice received bone marrow from either Lys-M-Cre×PPARγ^flox/flox^ mice or control mice (PPARγ^flox/flox^). Mice with targeted deletion of PPARγ in macrophages were generated by crossing PPARγ^flox/flox^ mice (C57BL/6 background; commercially available from JAX) with transgenic mice in which Cre recombinase is under the control of the M lysozyme promoter (Lys-M-Cre), similar to a previous report [34]. These mice, which are Lys-M-Cre^+/−^ PPARγ^flox/flox^ are designated PPARγ-Mac^neg^. For tumor studies with these mice, control mice used were PPARγ^flox/flox^.

### Cells

Stable clones of CMT/167 cells, derived from a spontaneous lung adenocarcinoma in C57BL/6 mice [13] expressing high levels of firefly luciferase constitutively driven by an SV40 promoter (CMT/167-luc), were used for injection into animals as described previously [5]. For bone marrow-derived macrophages, bone marrow–derived cells isolated from femurs and tibias of PPARγ^flox/flox^ or PPARγ-Mac^neg^ mice were cultured in the presence of 50 ng/mL M-CSF (R&D) to promote macrophage maturation as previously described [16]. After 5 days in culture, these cells have the morphology of macrophages, and are >95% F4/80 positive.

### Tumor cell injections

Mice were fed either control or pioglitazone-impregnated chow (0.05%) for 7 days prior to cancer cell injection and throughout the course of the experiment. This is an identical concentration used for rosiglitazone in the setting of experimental pulmonary hypertension [35]. By mass spectrometric analysis steady-state levels were approximately 5000 ng/mL (data not shown), which is somewhat higher than the 1500 ng/mL observed in human trials [36]. In an orthotopic model, mice were directly injected with CMT/167-luc cells (10^5^ per 40 µL) suspended in 1.35 mg/mL Matrigel Basement Membrane Matrix (BD Biosciences)/HBSS, through the rib cage into the left lobe of the lung using 30-g needles as described previously [5]. At time of sacrifice, mice were injected i.p. with 300 mg/kg body weight luciferin (Caliper) before sedation. The circulation was perfused and the lungs inflated with heparinized PBS. The heart and lungs were removed en bloc and the liver and brain were harvested for ex vivo bioluminescence imaging. The left lobe was isolated from the remaining lung lobes and imaged for bioluminescence separately; remaining lung lobes, heart, mediastinal lymph nodes, liver, and brain were also imaged separately using the IVIS Imaging System 50 Series (Caliper Life Sciences/Xenogen Corp.). Primary tumor size was measured using digital calipers and surface secondary pulmonary metastases were counted under a dissecting microscope. After imaging, all tissues were fixed with 4% paraformaldehyde (PFA) and embedded in paraffin for morphological and immunohistochemical analyses. Two additional methods were used to quantify secondary pulmonary metastases: 1) H&E stained lung sections were analyzed by counting all lobes for tumor formation; 3 sections per animal were counted by two blinded observers; 2) Ex vivo luminescence incidence was scored for each lung lobe. In addition, incidence of metastases to the liver and brain was scored by luminescence and verified by H&E staining.

As a second model, cancer cells were implanted into the flank subcutaneously as previously described [37]. Briefly, 10^5^ CMT/167-luc cells were injected as previously described [38]. Primary tumor size was measured as a function of time by digital calipers, and metastatic burden to the lungs was quantitated by examination of the lungs under a dissecting microscope or by bioluminescence.

### Immunofluorescence staining

For immunofluorescence labeling, PFA-fixed, paraffin-embedded tissues were deparaffinized, rehydrated, and underwent antigen retrieval by heating for 20 min at 115°C in a decloaking chamber (Biocare). Sections were then exposed to specific antibodies overnight at 4°C. After incubations with primary antibodies, antigen/antibody complexes were visualized using Alexa Fluor-568–coupled, Alexa Fluor-488–coupled, or Alexa-647-coupled secondary antibodies (Molecular Probes); for triple immunofluorescence, sections were sequentially incubated with specific primary and secondary antibodies. Coverslips were mounted with VectaShield medium containing 4′,6-diamidino-2-phenylindole to detect all cell nuclei (Vector Laboratories). Sections were visualized using a Nikon inverted fluorescence microscope equipped with Metamorph software or using a laser-scanning confocal microscope (510 META NLO, Carl Zeiss, Thornwood, NY) with LSM 510 software. Negative controls included the use of mouse or rabbit IgG. Antibodies used include polyclonal FITC-conjugated anti-GFP (1∶200; Abcam), monoclonal anti-Mac3 (1∶50; BD Pharmingen), and polyclonal anti-Arginase I (1∶200; Santa Cruz). Quantification of macrophage recruitment was determined by counting the number of Mac3-positive cells in 40× fields surrounding primary tumors or within tumors. Three 40× fields were counted per section; three sections per animal were counted.

### Measurement of mouse serum adiponectin levels

Eight-week old male C57BL/6 mice were fed either control chow or pioglitazone-impregnated chow. The mice were sacrificed either 7 or 23 days later, and serum was collected from each mouse. Serum adiponectin levels were measured by ELISA (R&D Systems Quantikine) as per manufacturer's protocol.

### *In vitro* co-culture

Bone marrow–derived macrophages (2×10^6^) from either WT, PPARγ^flox/flox^ or PPARγ-Mac^neg^ mice were isolated and grown on the bottom of 6-well plates for 4 days in the presence of M-CSF as previously described [5], [16]. CMT/167-luc cells (3×10^4^) were grown on Transwell filters containing 0.4 mm pores (Corning). Cells were placed in co-culture for 3 days, and lysates were prepared in RIPA buffer and immunoblotted for arginase I or iNOS. Arginase I antibody (Santa Cruz) was used at 1∶200, iNOS antibody (BD) was used at 1∶1000 and b-actin (Sigma) was used at 1∶5000 as a loading control. As a positive control macrophages were stimulated for 24 hours with 20 ng/ml interferon-g/100 ng/ml LPS to induce the M1 phenotype, and 20 ng/ml IL-4 (Sigma) to induce the M2 phenotype. Relative abundance of protein was determined by quantitative densitometry using ImageJ software (NIH, Bethesda, MD). All Western Blot densitometry data on arginase I were normalized to b-actin. The relative level of arginase I was then normalized by the level of arginase I in WT bone marrow-derived macrophages co-cultured with CMT/167-luc cells in the absence of pioglitazone.

### Statistical analyses

All values given represent mean values +/− SE. To compare the two groups, the Student's *t* and Mann-Whitney *U* tests were used (for normally or non-normally distributed data, respectively). ANOVA was used to detect significant differences between multiple groups. All *P* values are two-tailed; *P* values ≤0.05 were considered significant.

## Supporting Information

Figure S1 **Effects of Pioglitazone on CMT/167 Cells.** ***A.*** CMT/167 cells were plated on Matrigel-coated 8 mm Transwells, containing either 10 µM pioglitazone or vehicle (0.1% DMSO). After 48 hours, cells that had invaded through the pores were quantitated by DAPI staining. Pioglitazone decreased cell invasiveness; *P<0.05 vs Control. ***B.*** CMT/167 cells were grown in 3-dimensional Matrigel culture as previously described [10] in the absence of presence of 10 µM pioglitazone. Cells were fixed after 5 days, and regular spheroids, which are characteristic of differentiated cancer cells were quantitated. Images are representative of 5 independent fields at 100×. Graph at the bottom indicates quantitation. Pioglitazone increased the number of differentiated organoid structures; *P<0.05 vs Control.
(DOC)

Figure S2 **Rosiglitazone accelerates CMT/167 tumor progression and metastasis in a mouse flank model of NSCLC.** ***A.*** Volume of primary flank tumors from C57BL/6 mice fed either normal chow or chow impregnated with rosiglitazone (0.05%) at 28 days. ***B.*** Number of pulmonary metastases in C57BL/6 mice fed either normal chow or rosiglitazone chow at 28 days. ***C.*** Percentage of animals in each group with lung metastases at 28 days. *P<0.05 vs Control.
(DOC)

## References

[1] Jemal A, Siegel R, Ward E, Murray T, Xu J (2006). Cancer statistics.. CA Cancer J Clin.

[2] Kenny PA, Lee GY, Bissell MJ (2007). Targeting the tumor microenvironment.. Front Biosci.

[3] Johnson L, Mercer K, Greenbaum D, Bronson RT, Crowley D (2001). Somatic activation of the K-ras oncogene causes early onset lung cancer in mice.. Nature.

[4] Smith GJ, Le Mesurier SM, de Montfort ML, Lykke AW (1984). Establishment of epithelial cell strains from normal adult mouse lung resembling a urethane-induced lung adenoma cell strain and a metastasizing mouse lung carcinoma cell line.. Cell Biol Int Rep.

[5] Weiser-Evans MCM, Wang X-Q, Amin J, Van Putten V, Choudhary R (2009). Depletion of Cytosolic Phospholipase A2 in Bone Marrow-Derived Macrophages Protects against Lung Cancer Progression and Metastasis.. Cancer Res.

[6] Tontonoz P, Spiegelman BM (2008). Fat and beyond: the diverse biology of PPARgamma.. Annu Rev Biochem.

[7] Lehmann JM, Moore LB, Smith-Oliver TA, Wilkison WO, Willson TM (1995). An antidiabetic thiazolidinedione is a high affinity ligand for peroxisome proliferator-activated receptor gamma (PPAR gamma).. J Biol Chem.

[8] Sasaki H, Tanahashi M, Yukiue H, Moiriyama S, Kobayashi Y (2002). Decreased perioxisome proliferator-activated receptor gamma gene expression was correlated with poor prognosis in patients with lung cancer.. Lung Cancer.

[9] Govindarajan R, Ratnasinghe L, Simmons DL, Siegel ER, Midathada MV (2007). Thiazolidinediones and the Risk of Lung, Prostate, and Colon Cancer in Patients With Diabetes.. J Clin Oncol.

[10] Bren-Mattison Y, Meyer AM, Van Putten V, Li H, Kuhn K (2008). Antitumorigenic Effects of Peroxisome Proliferator-Activated Receptor-{gamma} in Non-Small-Cell Lung Cancer Cells Are Mediated by Suppression of Cyclooxygenase-2 via Inhibition of Nuclear Factor-{kappa}B.. Mol Pharmacol.

[11] Bren-Mattison Y, Van Putten V, Chan D, Winn R, Geraci MW (2005). Peroxisome proliferator-activated receptor-gamma (PPAR(gamma)) inhibits tumorigenesis by reversing the undifferentiated phenotype of metastatic non-small-cell lung cancer cells (NSCLC).. Oncogene.

[12] Nemenoff R, Meyer AM, Hudish TM, Mozer AB, Snee A (2008). Prostacyclin Prevents Murine Lung Cancer Independent of the Membrane Receptor by Activation of Peroxisomal Proliferator-Activated Receptor {gamma}.. Cancer Prev Res.

[13] Franks LM, Carbonell AW, Hemmings VJ, Riddle PN (1976). Metastasizing tumors from serum-supplemented and serum-free cell lines from a C57BL mouse lung tumor.. Cancer Res.

[14] Maeda N, Takahashi M, Funahashi T, Kihara S, Nishizawa H (2001). PPARgamma ligands increase expression and plasma concentrations of adiponectin, an adipose-derived protein.. Diabetes.

[15] Mantovani A, Marchesi F, Porta C, Sica A, Allavena P (2007). Inflammation and cancer: breast cancer as a prototype.. Breast.

[16] Riches DW, Henson PM, Remigio LK, Catterall JF, Strunk RC (1988). Differential regulation of gene expression during macrophage activation with a polyribonucleotide. The role of endogenously derived IFN.. J Immunol.

[17] Mantovani A, Sozzani S, Locati M, Allavena P, Sica A (2002). Macrophage polarization: tumor-associated macrophages as a paradigm for polarized M2 mononuclear phagocytes.. Trends Immunol.

[18] Han SW, Roman J (2008). Activated PPARgamma Targets Surface and Intracellular Signals That Inhibit the Proliferation of Lung Carcinoma Cells.. PPAR Res.

[19] Li M, Lee TW, Yim AP, Mok TS, Chen GG (2006). Apoptosis induced by troglitazone is both peroxisome proliferator-activated receptor-gamma- and ERK-dependent in human non-small lung cancer cells.. J Cell Physiol.

[20] Zou W, Liu X, Yue P, Khuri FR, Sun SY (2007). PPARgamma ligands enhance TRAIL-induced apoptosis through DR5 upregulation and c-FLIP downregulation in human lung cancer cells.. Cancer Biol Ther.

[21] Choudhary R, Li H, Winn RA, Sorenson AL, Weiser-Evans MC (2009). Peroxisome Proliferator-Activated Receptor-gamma Inhibits Transformed Growth of Non-Small Cell Lung Cancer Cells through Selective Suppression of Snail.. Neoplasia.

[22] Li MY, Yuan H, Ma LT, Kong AW, Hsin MK (2010). Roles of peroxisome proliferator-activated receptor-alpha and -gamma in the development of non-small cell lung cancer.. Am J Respir Cell Mol Biol.

[23] Lewis CE, Pollard JW (2006). Distinct Role of Macrophages in Different Tumor Microenvironments.. Cancer Res.

[24] Rodriguez PC, Quiceno DG, Zabaleta J, Ortiz B, Zea AH (2004). Arginase I production in the tumor microenvironment by mature myeloid cells inhibits T-cell receptor expression and antigen-specific T-cell responses.. Cancer Res.

[25] Liu Q, Zhang C, Sun A, Zheng Y, Wang L (2009). Tumor-educated CD11bhighIalow regulatory dendritic cells suppress T cell response through arginase I.. J Immunol.

[26] Norian LA, Rodriguez PC, O'Mara LA, Zabaleta J, Ochoa AC (2009). Tumor-infiltrating regulatory dendritic cells inhibit CD8+ T cell function via L-arginine metabolism.. Cancer Res.

[27] Bak SP, Alonso A, Turk MJ, Berwin B (2008). Murine ovarian cancer vascular leukocytes require arginase-1 activity for T cell suppression.. Mol Immunol.

[28] Oberlies J, Watzl C, Giese T, Luckner C, Kropf P (2009). Regulation of NK cell function by human granulocyte arginase.. J Immunol.

[29] Gupta RA, Dubois RN (2002). Controversy: PPARgamma as a target for treatment of colorectal cancer.. Am J Physiol Gastrointest Liver Physiol.

[30] Lefebvre AM, Chen I, Desreumaux P, Najib J, Fruchart JC (1998). Activation of the peroxisome proliferator-activated receptor gamma promotes the development of colon tumors in C57BL/6J-APCMin/+ mice.. Nat Med.

[31] Pino MV, Kelley MF, Jayyosi Z (2004). Promotion of colon tumors in C57BL/6J-APC(min)/+ mice by thiazolidinedione PPARgamma agonists and a structurally unrelated PPARgamma agonist.. Toxicol Pathol.

[32] Girnun GD, Smith WM, Drori S, Sarraf P, Mueller E (2002). APC-dependent suppression of colon carcinogenesis by PPARgamma.. Proc Natl Acad Sci U S A.

[33] Nemenoff RA (2007). Peroxisome proliferator-activated receptor-gamma in lung cancer: defining specific versus “off-target” effectors.. J Thorac Oncol.

[34] Bonfield TL, Thomassen MJ, Farver CF, Abraham S, Koloze MT (2008). Peroxisome Proliferator-Activated Receptor-{gamma} Regulates the Expression of Alveolar Macrophage Macrophage Colony-Stimulating Factor.. J Immunol.

[35] Crossno JT, Garat CV, Reusch JEB, Morris KG, Dempsey EC (2007). Rosiglitazone attenuates hypoxia-induced pulmonary arterial remodeling.. Am J Physiol Lung Cell Mol Physiol.

[36] Budde K, Neumayer HH, Fritsche L, Sulowicz W, Stompor T (2003). The pharmacokinetics of pioglitazone in patients with impaired renal function.. Br J Clin Pharmacol.

[37] Heasley LE, Thaler S, Nicks M, Price B, Skorecki K (1997). Induction of cytosolic phospholipase A_2_ by oncogenic Ras in human non-small cell lung cancer.. J Biol Chem.

[38] Wick M, Hurteau G, Dessev C, Chan D, Geraci MW (2002). Peroxisome Proliferator-Activated Receptor-gamma Is a Target of Nonsteroidal Anti-Inflammatory Drugs Mediating Cyclooxygenase-Independent Inhibition of Lung Cancer Cell Growth.. Mol Pharmacol.

