# Supplementary figures and images for: Activation of PPARγ in Myeloid Cells Promotes Lung Cancer Progression and Metastasis
Source: PLoS One. 2011 Dec 1;6(12):e28133. doi: 10.1371/journal.pone.0028133 (PMC3228753; doi:10.1371/journal.pone.0028133)

| 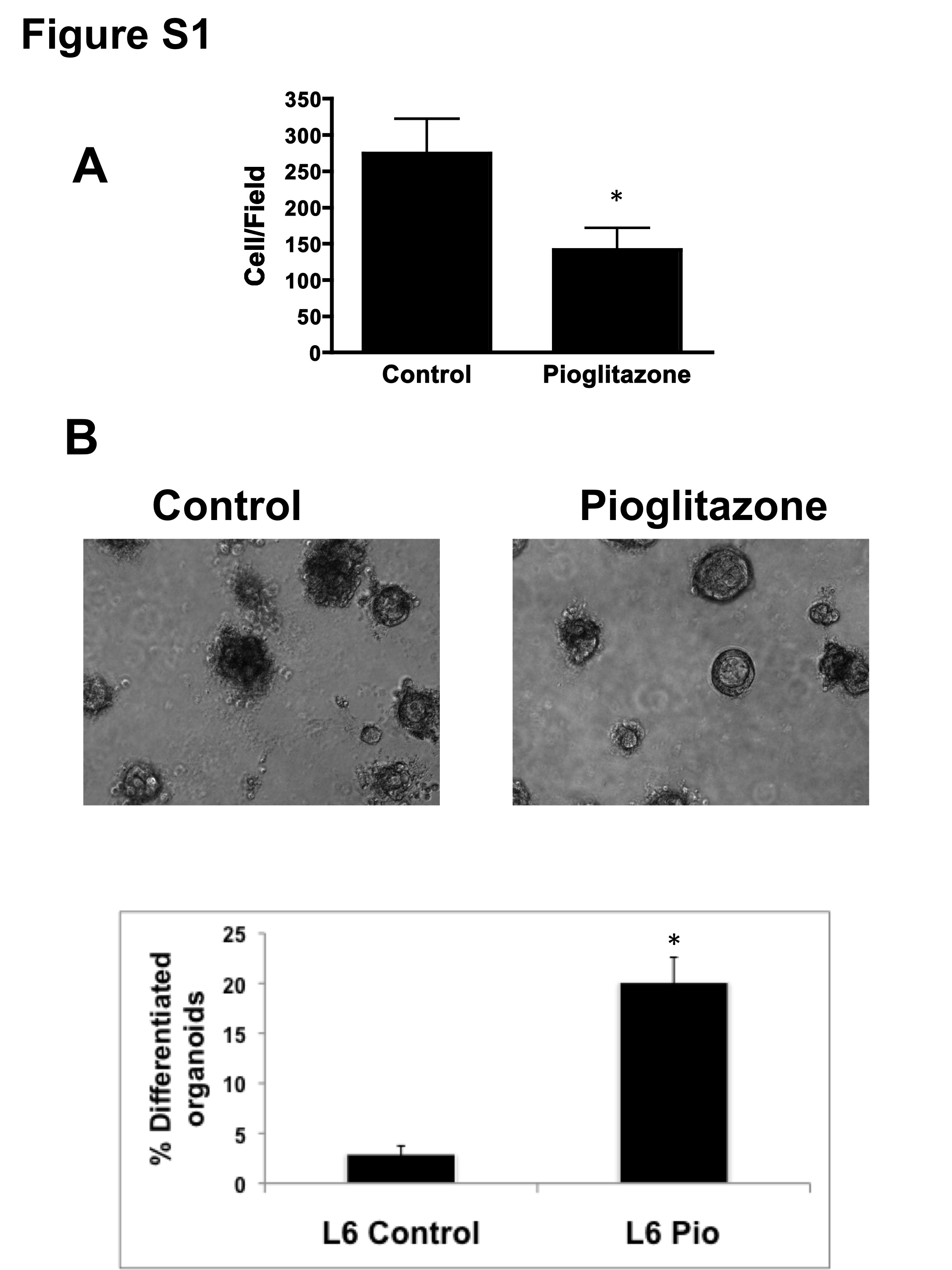 |
| --- |

Supplement: Figure S1 — Effects of Pioglitazone on CMT/167 Cells. A. CMT/167 cells were plated on Matrigel-coated 8 mm Transwells, containing either 10 µM pioglitazone or vehicle (0.1% DMSO). After 48 hours, cells that had invaded through the pores were quantitated by DAPI staining. Pioglitazone decreased cell invasiveness; *P<0.05 vs Control. B. CMT/167 cells were grown in 3-dimensional Matrigel culture as previously described [10] in the absence of presence of 10 µM pioglitazone. Cells were fixed after 5 days, and regular spheroids, which are characteristic of differentiated cancer cells were quantitated. Images are representative of 5 independent fields at 100×. Graph at the bottom indicates quantitation. Pioglitazone increased the number of differentiated organoid structures; *P<0.05 vs Control. (DOC) [file pone.0028133.s001.doc]

| **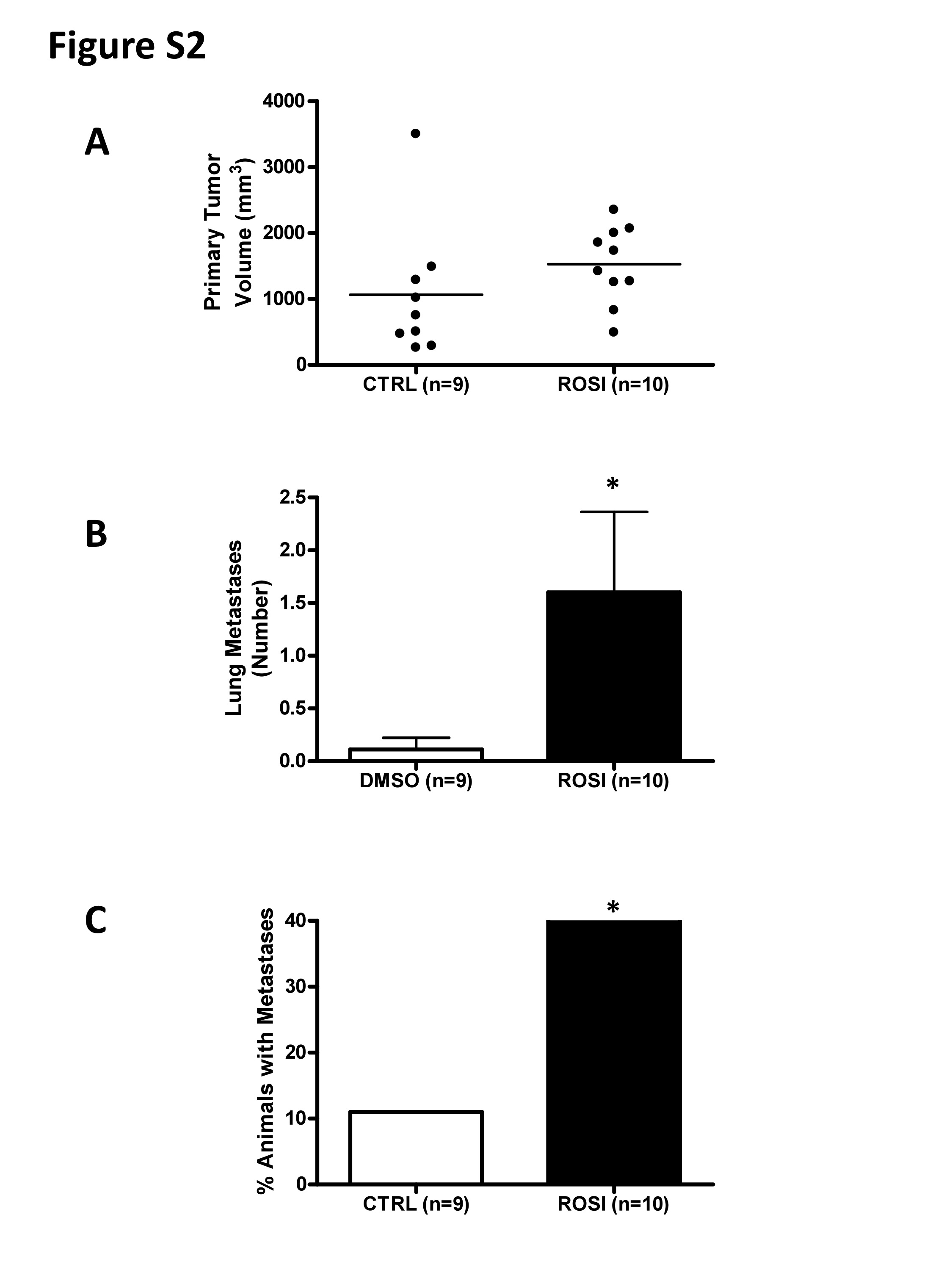** |
| --- |

Supplement: Figure S2 — Rosiglitazone accelerates CMT/167 tumor progression and metastasis in a mouse flank model of NSCLC. A. Volume of primary flank tumors from C57BL/6 mice fed either normal chow or chow impregnated with rosiglitazone (0.05%) at 28 days. B. Number of pulmonary metastases in C57BL/6 mice fed either normal chow or rosiglitazone chow at 28 days. C. Percentage of animals in each group with lung metastases at 28 days. *P<0.05 vs Control. (DOC) [file pone.0028133.s002.doc]
